# Acoustic Source Characteristics, Across-Formant Integration, and Speech Intelligibility Under Competitive Conditions

**DOI:** 10.1037/xhp0000038

**Published:** 2015-03-09

**Authors:** Brian Roberts, Robert J. Summers, Peter J. Bailey

**Affiliations:** 1 Psychology, School of Life and Health Sciences, Aston University; 2 Department of Psychology, University of York

**Keywords:** speech intelligibility, formant integration, informational masking, auditory grouping, source characteristics

## Abstract

An important aspect of speech perception is the ability to group or select formants using cues in the acoustic source characteristics—for example, fundamental frequency (F0) differences between formants promote their segregation. This study explored the role of more radical differences in source characteristics. Three-formant (F1+F2+F3) synthetic speech analogues were derived from natural sentences. In Experiment 1, F1+F3 were generated by passing a harmonic glottal source (F0 = 140 Hz) through second-order resonators (H1+H3); in Experiment 2, F1+F3 were tonal (sine-wave) analogues (T1+T3). F2 could take either form (H2 or T2). In some conditions, the target formants were presented alone, either monaurally or dichotically (left ear = F1+F3; right ear = F2). In others, they were accompanied by a competitor for F2 (F1+F2C+F3; F2), which listeners must reject to optimize recognition. Competitors (H2C or T2C) were created using the time-reversed frequency and amplitude contours of F2. Dichotic presentation of F2 and F2C ensured that the impact of the competitor arose primarily through informational masking. In the absence of F2C, the effect of a source mismatch between F1+F3 and F2 was relatively modest. When F2C was present, intelligibility was lowest when F2 was tonal and F2C was harmonic, irrespective of which type matched F1+F3. This finding suggests that source type and context, rather than similarity, govern the phonetic contribution of a formant. It is proposed that wideband harmonic analogues are more effective informational maskers than narrowband tonal analogues, and so become dominant in across-frequency integration of phonetic information when placed in competition.

The production of speech is usually characterized in terms of source-filter theory (Fant, 1960; Stevens, 1998), in which the acoustic signal is shaped by passing the sounds arising from one or more excitation sources (mainly voicing, frication, and plosion) through the air-filled cavities of the talker’s vocal tract. The vocal tract acts as a filter—components of the source close to one of its resonant frequencies are emphasized, giving rise to spectral prominences in the speech signal known as formants. These formants are important perceptually because they convey articulatory information about the shape of the vocal tract and how it changes over time as the tongue, lips, and jaw are moved by the talker. Hence, listeners trying to understand a spoken message benefit considerably from the information carried by formant frequencies and their change over time (e.g., Roberts, Summers, & Bailey, 2011). Precisely how information carried by different formants is integrated across frequency into a phonetic percept is not fully understood, especially in contexts where more than one person is speaking at once (e.g., Darwin, 2008).

The talker has considerable scope for independent control of the source and filter characteristics of speech—for example, producing the same syllable on a different pitch or on a whisper—and so a distinction can be made between the abstract phonetic information carried by the time-varying formants and the acoustic form in which they are rendered. For example, intelligible analogues of speech can be generated using only a buzz excitation source, even when the synthetic version contains segments corresponding to unvoiced fricatives and plosives (e.g., Summers, Bailey, & Roberts, 2010, 2012). In principle, useful information about formant-frequency variation could be carried by a wide variety of source characteristics, extending well beyond those that might plausibly be produced by a human talker. Most notably, intelligible sine-wave speech can be synthesized despite the radical simplifications involved in replacing the acoustic richness of each formant in natural speech with a single time-varying sinusoid that tracks the frequency and amplitude contour of that formant (Bailey, Summerfield, & Dorman, 1977; Remez, Rubin, Pisoni, & Carrell, 1981).

Several factors are likely to influence the impact of acoustic source characteristics on the ability of listeners to extract information from a formant, and to form a phonetic percept by integrating this information across formants. For example, it has long been known that the intelligibility of sine-wave speech is lower than otherwise comparable harmonic analogues (Bailey et al., 1977; Remez et al., 1981). Nonetheless, selecting the right set of formants from the speech mixture is always critical for intelligibility when there is more than one talker. The current study explores the effect of whether or not: (a) all formant analogues comprising the target utterance are synthesized using the same source characteristics; (b) analogues of extraneous formants are also present. These two factors are likely to interact, as it is well-established that the factors governing perceptual organization are generally revealed most clearly where competition operates (e.g., Barker & Cooke, 1999; Darwin, 1981).

To date, the ability of listeners to use cues in the source characteristics to group and select formants has been examined mainly in the context of fundamental frequency (F0). Several studies in which the different formants of voiced speech are presented on the same or different pitches have shown that differences in F0 can increase identification accuracy for pairs of concurrent vowels (e.g., Assmann & Summerfield, 1994; Summerfield & Assmann, 1991), influence the identity of consonant-vowel (CV) syllables (Darwin, 1981; Gardner, Gaskill, & Darwin, 1989), and raise the intelligibility of longer utterances (Bird & Darwin, 1998; Summers et al., 2010). In the current study, we investigated the effect of introducing more radical acoustic differences between formant analogues. Some analogues were generated by passing a harmonic source through a second-order resonator; others were rendered as tonal (sine-wave) analogues. Beyond studies of duplex perception, in which only the distinguishing formant transition in a synthetic CV syllable is replaced by a sinusoidal glide (e.g., Bailey & Herrmann, 1993; Whalen & Liberman, 1987), few studies have used hybrid stimuli of this type. A rare exception is a study in which isolated steady vowels whose formants were either all-harmonic or a mixture of harmonic and sine-wave analogues were used to compare the extent to which young children and adults integrate spectrally disparate elements in a speech signal (Nittrouer & Tarr, 2011). However, to our knowledge, no studies have used sentence-length materials of this type or compared the effect of an across-formant mismatch in source characteristics in target-only and target-plus-interferer contexts.

The experiments reported here used the second-formant competitor (F2C) paradigm, in which listeners must reject a single extraneous formant to optimize recognition of short sentences (Remez, Rubin, Berns, Pardo, & Lang, 1994; Roberts, Summers, & Bailey, 2010, 2014; Summers et al., 2010). Central to this paradigm is the dichotic presentation of the target F2 and F2C, which may be considered as an alternative candidate for the second formant. In the version of the paradigm used here, the first and third formants (F1 and F3) are presented in the ear receiving F2C. Note that the lateralization cues for this configuration favor the fusion of F1+F3 with F2C rather than with the target F2 (cf. Culling & Summerfield, 1995), and so might be expected to increase the impact of the competitor formant on intelligibility. Moreover, this configuration greatly reduces energetic masking of the target formants—that is, masking caused by the energy of the competitor formant swamping that of the target formants (especially F2) in the auditory-nerve response to the stimulus. Hence, the interference caused by the extraneous formant must arise primarily through informational masking—an effect of central origin that may arise from a failure of auditory object formation, incorrect object selection, or general limits on processing capacity (see, e.g., Durlach, Mason, Kidd et al., 2003; Kidd, Mason, Richards, Gallun, & Durlach, 2008; Shinn-Cunningham, 2008). Note that the impact of interfering speech on the intelligibility of attended speech often arises mainly through informational masking (e.g., Brungart, Chang, Simpson, & Wang, 2006). This is because speech is a sparse signal on a frequency-time representation—it consists mainly of discrete harmonics whose amplitudes peak near the formant frequencies, and it contains silent or low-amplitude portions associated with closures. Hence, when two speech signals of similar level are mixed together, each local frequency-time region is usually dominated by one or the other of the two signals. This means that separating two voices is often mainly a problem of assigning readily detectable frequency-time regions to the correct source rather than one of detecting parts of the target signal (see, e.g., Darwin, 2008).

Several studies using multitone maskers configured to minimize energetic masking have shown that informational masking can be reduced by including grouping cues that assist the perceptual segregation of the target from the masker. For example, substantial release from informational masking can be obtained in detection and discrimination tasks when a spatial separation is introduced between target and masker (e.g., Arbogast, Mason, & Kidd, 2002; Kidd, Mason, Deliwala, Woods, & Colburn, 1994), or when the masker begins before the target (e.g., Hall, Buss, & Grose, 2005). The benefits of segregation cues have also been found in suprathreshold contexts where the target must be recognized, such as identifying distinctive arbitrary patterns (Kidd, Mason, Rohtla, & Deliwala, 1998) or the calls of songbirds (Best, Ozmeral, Gallun, Sen, & Shinn-Cunningham, 2005). Furthermore, speech recognition studies have shown that even an illusory spatial separation between target and masker—induced by the precedence effect (see, e.g., Zurek, 1987)—can reduce informational masking of target speech by interfering speech while leaving energetic masking unaffected (e.g., Freyman, Balakrishnan, & Helfer, 2001; Freyman, Helfer, McCall, & Clifton, 1999).

Of particular relevance to the current study, qualitative differences between target and masker also reduce informational masking. For example, it is easier to detect a narrowband-noise target than a tonal target in a multitone masker (e.g., Neff, 1995) or to detect a target tone with a frequency sweep in the opposite direction to that of the masking tones (Durlach, Mason, Shinn-Cunningham et al., 2003). Furthermore, there is evidence of a negative correlation between the ability of listeners to detect a target embedded in a masker and their judgments of target-masker similarity when target and masker are presented separately (Lee & Richards, 2011). Of course, there are many dimensions along which targets and maskers can differ perceptually, and not all of them necessarily act as grouping constraints. The multitone masker studies considered above all made use of relatively simple qualitative differences between target and masker, whereas studies using speech analogues and involving across-formant differences in dynamic properties suggest that these differences provide little or no basis for the grouping and segregation of formants. For example, adding formant transitions to one member of a pair of concurrent vowels provides only a small benefit in identification accuracy (Assmann, 1995, 1996). Furthermore, listeners seem unable to use differences in the rate or depth of formant-frequency variation to segregate an extraneous formant from the formants ensemble comprising a target sentence (Roberts et al., 2014; Summers et al., 2012). Note, however, that differences in acoustic source characteristics between formant analogues are similar conceptually to the simple qualitative differences typically used in the multitone masker studies. On that basis, one might predict that across-formant integration would be facilitated when there is a match in source characteristics but hindered when there is a mismatch.

## Experiment 1

In this experiment, F1 and F3 were always generated by passing a monotonous periodic source through second-order resonators. The target F2 was either generated the same way or was a sine-wave (tonal) analogue. When present, the extraneous competitor (F2C) could also take either form. This approach allowed us to explore the effects of matches and mismatches in acoustic source characteristics within formant ensembles, both in target-only and target-plus-interferer listening contexts. Although there are inevitably differences in the amount of phonetic information carried by harmonic and tonal analogues of formants with the same frequency and amplitude contours, this can be controlled for by making an appropriate choice of comparisons across conditions.

### Method

#### Listeners

Volunteers were first tested using a screening audiometer (Interacoustics AS208, Assens, Denmark) to ensure that their audiometric thresholds at 0.5, 1, 2, and 4 kHz did not exceed 20 dB HL. All volunteers who passed the audiometric screening took part in a training session designed to improve the intelligibility of the speech analogues used (see Procedure). About two thirds of these volunteers completed the training successfully and took part in the main experiment. All of these listeners met the additional criterion of a mean score of ≥20% keywords correct in the main experiment, when collapsed across all conditions, and so their results were included in the final dataset. This nominally low criterion was chosen to take into account the poor intelligibility expected for some of the stimulus materials used. Twenty-four listeners (5 males) successfully completed the experiment (mean age = 21.4 years, range = 18.9–31.0). To our knowledge, none of the listeners had heard any of the sentences used in the main part of the experiment in any previous study or assessment of their speech perception. All listeners were native speakers of English and gave informed consent. The research was approved by the Aston University Ethics Committee.

#### Stimuli and conditions

The stimuli for the main experiment were derived from recordings of the Bamford-Kowal-Bench (BKB) sentence lists (Bench, Kowal, & Bamford, 1979), spoken by a British male talker of “Received Pronunciation” English. To enhance the intelligibility of the synthetic analogues, the 48 sentences used were semantically simple and selected to contain ≤25% phonemes involving vocal tract closures or unvoiced frication. A set of keywords was chosen for each sentence; most designated keywords were content words. The stimuli for the training session were derived from 40 sentences spoken by a different talker and taken from commercially available recordings of the Harvard sentence lists (Institute of Electrical and Electronics Engineers, 1969). These sentences were also selected to contain ≤25% phonemes involving closures or unvoiced frication.

For each sentence, the pitch contour and the frequency contours of the first three formants were estimated from the waveform automatically every 1 ms from a 25-ms-long Gaussian window, using custom scripts in Praat (Boersma & Weenink, 2010). In practice, the third-formant contour often corresponded to the fricative formant rather than F3 during phonetic segments with frication; these cases were not treated as errors. Gross errors in automatic estimates of the three formant frequencies were hand-corrected using a graphics tablet; artifacts are not uncommon and manual post-processing of the extracted formant tracks is often necessary (Remez et al., 2011). Amplitude contours corresponding to the corrected formant frequencies were extracted automatically from the stimulus spectrograms; these frequency and amplitude contours were used to generate synthetic analogues of each sentence.

In all conditions, the frequency and amplitude contours of F1 and F3 were used to control two parallel buzz-excited second-order resonators. Hence, F1+F3 provided a common “harmonic frame” shared by all conditions. A monotonous source (F0 = 140 Hz) was used in the synthesis of all buzz-excited materials in the training and main session of the experiment. The excitation source was a periodic train of pulses modeled on the glottal waveform, shown by Rosenberg (1971) to be capable of producing synthetic speech of good quality. In the all-harmonic target conditions (H1+H2+H3), the frequency and amplitude contours of F2 were used to control a third parallel buzz-excited resonator. The 3-dB bandwidths of the resonators corresponding to F1, F2, and F3 were set to constant values of 50, 70, and 90 Hz, respectively. In the hybrid-target conditions (H1+T2+H3), the frequency and amplitude contours of F2 were instead used to control the properties of a time-varying sinusoid. This tonal analogue of F2 (T2) was matched to the root mean square (RMS) power of its harmonic counterpart (H2) before being combined with the harmonic F1+F3 frame.

For each sentence in the main experiment, second-formant competitors (F2Cs) were generated using the time-reversed frequency and amplitude contours of the corresponding target F2; this manipulation preserves the rate and depth of frequency variation found in the F2 contour. These competitors were rendered either as the output of a buzz-excited resonator (H2C) or as an RMS-matched time-varying sinusoid (T2C). In the former case, the excitation source (Rosenberg pulses), F0 frequency (140 Hz), and 3-dB bandwidth (70 Hz) were identical to those used to synthesize the target F2. The waveform of the excitation source for H2C was not time reversed. When present, F2C was always delivered to the ear receiving F1+F3. Stimuli were selected such that the frequency of F2C was always ≥80 Hz from the frequencies of F1 and F3 at any moment in time. Hence, within the same ear, there were no crossovers of formant tracks or any approaches close enough to cause audible interactions between corresponding harmonics exciting adjacent formants. Following Klatt (1980), the outputs of the resonators corresponding to F1, F2/F2C, and F3 were summed using alternating signs (+, –, +) to minimize spectral notches between adjacent formants.

**Figure 1 fig1:**
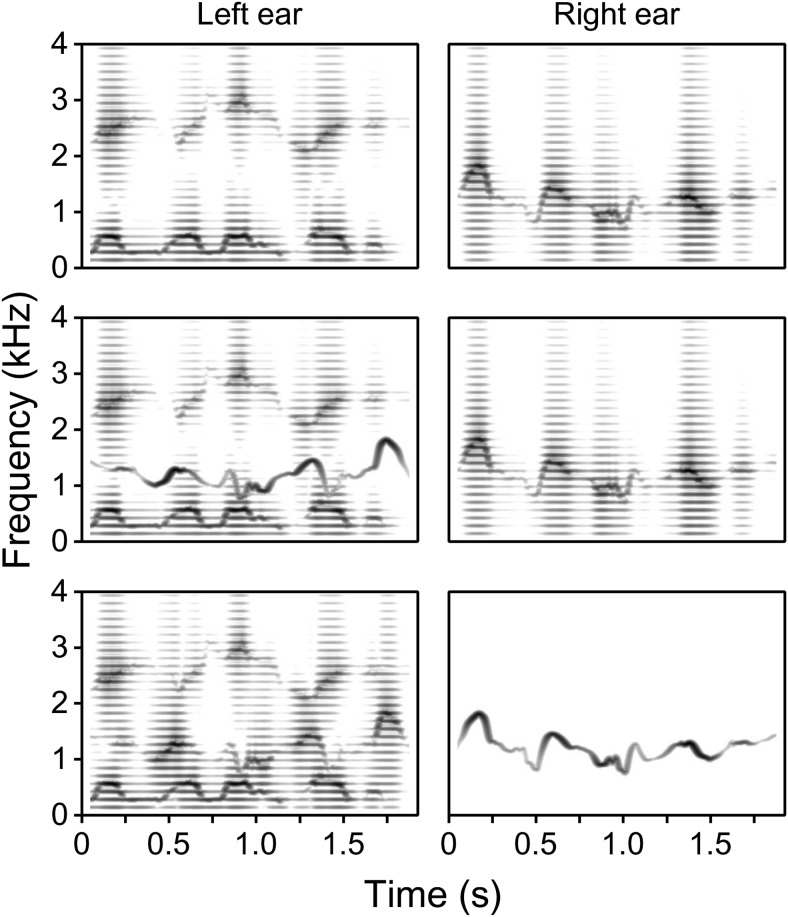
Stimuli for Experiment 1—stimulus spectrograms illustrating three of the dichotic configurations used, derived by narrow-band frequency analysis of synthetic analogues of the example sentence “Men wear long trousers.” The left ear receives F1, F3, and (when present) F2C; the right ear receives the target F2. Formants are rendered either as harmonic (H) or tonal (T) analogues. The F1+F3 frame was harmonic in all conditions; the F0 frequency of harmonic analogues was 140 Hz. The upper, middle, and lower panels illustrate the stimulus configurations (H1+H3; H2), (H1+T2C+H3; H2), and (H1+H2C+H3; T2), respectively. The frequency and amplitude contours of F2C (when present) were time-reversed with respect to the target F2.

There were eight conditions in the main experiment (see Table 1). The stimuli for C1 comprised the F1+F3 frame and F2C (harmonic version), but the target F2 was absent. The stimuli for C2–C4 comprised all three target formants plus the competitor in a dichotic configuration (F1+F2C+F3; F2). This set represents three of the four possible combinations of acoustic properties for F2 and F2C; the case where both F2 and F2C are tonal analogues, and so neither matches the harmonic F1+F3 frame, was not included. The stimuli for the remaining conditions comprised only the target formants, presented either dichotically (C5–C6) or monaurally (C7–C8). Figure 1 illustrates three of the stimulus configurations used—those for C5 (H1+H3; H2) in the top panels, C3 (H1+T2C+H3; H2) in the middle panels, and C4 (H1+H2C+H3; T2) in the bottom panels. For each listener, the 48 sentences were divided equally across conditions (i.e., 6 per condition), such that there were always 18 or 19 keywords per condition. Allocation of sentences was counterbalanced by rotation across each set of 8 listeners tested. Hence, the total number of listeners needed to produce a balanced dataset was a multiple of 8.[Table-anchor tbl1][Fig-anchor fig1]

#### Procedure

During testing, listeners were seated in front of a computer screen and a keyboard in a sound-attenuating chamber (Industrial Acoustics 1201A, Winchester, United Kingdom). The experiment consisted of a training session followed by the main session and took about 45–60 min to complete; listeners were free to take a break whenever they wished. In both parts of the experiment, stimuli were presented in a new quasi-random order for each listener.

The training session comprised 50 trials; stimuli were presented diotically without competitors, and a new sentence was used for each trial. Half of the sentences were rendered as all-harmonic analogues (H1+H2+H3), and the others differed only in that F2 was instead rendered as a sine-wave analogue (H1+T2+H3). On each of the first 10 trials, participants heard the synthetic version (S) and the original recording (clear, C) of a given sentence in the order SCSCS; no response was required, but participants were asked to listen to these sequences carefully. On each of the remaining trials, listeners first heard the synthetic version of a given sentence, which they were asked to transcribe using the keyboard. They were allowed to listen to the stimulus up to a maximum of 6 times before typing in their transcription. After each transcription was entered, feedback to the listener was provided by playing the original recording (44.1 kHz sample rate) followed by a repeat of the synthetic version. Davis, Johnsrude, Hervais-Adelman, Taylor, and McGettigan (2005) found this strategy to be an efficient way of enhancing the perceptual learning of speech-like stimuli. A mean criterion of ≥50% keywords correct across the last 20 training trials was set for a listener to continue on to the main session. In the main session, as for training, participants were able to listen to each stimulus up to 6 times without time limit before typing in their transcription. However, in the main session, they did not receive feedback of any kind on their responses.

All speech analogues were synthesized using Mitsyn (Henke, 2005) at a sample rate of 22.05 kHz and with 10-ms raised-cosine onset and offset ramps. They were played at 16-bit resolution over Sennheiser HD 480-13II earphones (Hannover, Germany) via a Santa Cruz sound card (Turtle Beach, Valhalla, New York), programmable attenuators (Tucker-Davis Technologies PA5, Alachua, Florida), and a headphone buffer (TDT HB7). Output levels were calibrated using a sound-level meter (Brüel & Kjaer, Type 2209, Nærum, Denmark) coupled to the earphones by an artificial ear (Type 4153). Stimuli in the main experiment were presented at a reference level (long-term average) of 75 dB SPL; this describes the case when the left ear receives F1 (the most intense formant) and F3. For a given sentence, F1 and F3 were presented at the reference level in all conditions; hence there was some variation in the overall level and loudness of the stimuli across conditions depending on the presence or absence of F2 and F2C (and the ears receiving them). In the training session, the presentation level of the original recordings and speech analogues was lowered to 72 dB SPL, roughly to offset the increased loudness arising from binaural summation.

#### Data analysis

For each listener, the intelligibility of each stimulus was quantified in terms of the percentage of keywords identified correctly; homonyms were accepted. The stimuli for each condition comprised 6 sentences. Given the variable number of keywords per sentence (3 or 4), the mean score for each listener in each condition was computed as the percentage of keywords reported correctly giving equal weight to all the keywords used. Following the procedure of Roberts et al. (2010), we classified responses using tight scoring, in which a response is scored as correct only if it matches the keyword exactly (see Foster et al., 1993). All statistical analyses reported here were computed using SPSS (SPSS statistics version 20, IBM). The measure of effect size reported here is partial eta squared (η_p_^2^).

### Results

**Figure 2 fig2:**
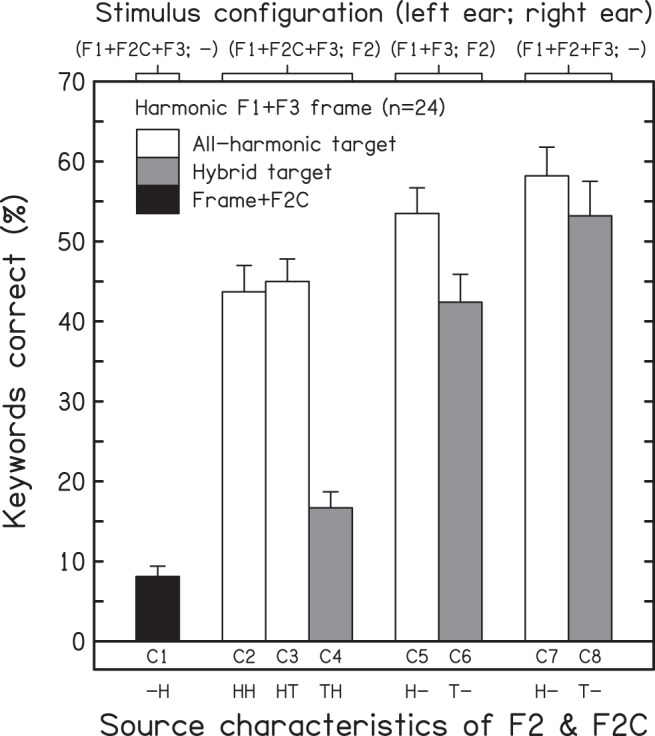
Results for Experiment 1—influence of source characteristics on the effect of competitors (F2Cs) on the intelligibility of synthetic analogues of sentences when the F1+F3 frame was harmonic. Mean scores and intersubject standard errors (*n* = 24) are shown for the frame+F2C condition (black bar), the conditions for which all formants of the target speech are harmonic analogues (matched, white bars), and the conditions for which the target is a hybrid comprising the harmonic F1+F3 frame and a tonal analogue of F2 (mismatched, gray bars). The top axis indicates which formants were presented to each ear; the bottom axis indicates the source characteristics of F2 and F2C—harmonic (H) or tonal (T). The frequency and amplitude contours of F2C (when present) were time-reversed with respect to the target F2.

Figure 2 shows the mean percentage scores (and intersubject standard errors) across conditions for keyword identification. The black, white, and gray bars indicate the results for the frame+F2C, all-harmonic target, and hybrid-target conditions, respectively. A one-way within-subjects analysis of variance (ANOVA) showed a highly significant effect of condition on intelligibility, *F*(7, 161) = 40.187, *p* < .001, η_p_^2^ = 0.636. Given the limited number of pairwise comparisons (two-tailed) required from the large set of possible comparisons, they were computed using the restricted least significant difference test (Keppel, 1991; Snedecor & Cochran, 1967). Condition C1 (H1+H2C+H3; –) indicated that intelligibility was near floor when F2C was added in the absence of the target F2. Pairwise comparisons indicated that the mean for C1 differed from those for all other conditions (*p* < .001, in all cases). Clearly, F2C was not a good surrogate for the target F2 in supporting intelligibility. Performance was best when the target formants were presented monaurally and without competitors (C7 and C8).[Fig-anchor fig2]

A one-way ANOVA restricted to the five dichotic cases (C2–C6) also showed a highly significant effect of condition, *F*(4, 92) = 25.587, *p* < .001, η_p_^2^ = 0.527. Adding an F2C to the target formants typically reduced intelligibility, but the extent of competitor impact depended on the source characteristics of F2 and of F2C. Competitor impact was explored using three pairwise comparisons. Adding either a harmonic or tonal F2C to a stimulus containing H2 (i.e., all-harmonic target) caused a modest but significant fall in intelligibility, C2 versus C5 = 9.8 percentage points, *t*(23) = 2.28, *p* = .032; C3 versus C5 = 8.5 percentage points, *t*(23) = 2.18, *p* = .040. Adding a harmonic F2C to a stimulus containing T2 (i.e., hybrid target) caused a substantial fall in intelligibility, C4 versus C6 = 25.7 percentage points, *t*(23) = 6.05, *p* < .001. Using the difference scores to compare the impact of adding H2C when F2 was rendered as either T2 or H2 showed that the two cases were significantly different, (C5–C2) versus (C6–C4) = 15.9 percentage points, *t*(23) = 2.62, *p* = .015.

The effect of differences in the acoustic form of the target F2 (harmonic vs. tonal) and of its ear of presentation (monaural vs. dichotic) was explored using a two-way ANOVA restricted to the F2C-absent conditions (C5–C8). This analysis revealed significant main effects of F2 source characteristics, *F*(1, 23) = 5.312, *p* = .031, η_p_^2^ = 0.188, and of the ear receiving F2, *F*(1, 23) = 5.625, *p* = .026, η_p_^2^ = 0.197, but these factors did not interact, *F*(1, 23) = 0.797, *p* = .381. When the target formants were presented alone, the intelligibility cost of introducing a source mismatch between F2 and the harmonic F1+F3 frame (*M* = 8.1 percentage points) was relatively modest, and so too was the cost of dichotic presentation (*M* = 7.7 percentage points).

### Discussion

When only the target formants are present, the intelligibility cost of presenting F1+F3 and F2 to opposite ears is broadly similar to that observed in our previous studies (Roberts et al., 2010; Summers et al., 2010). Sentence intelligibility is typically reduced when the target speech is accompanied by a competitor formant (F2C) created using the time-reversed frequency and amplitude contours of F2 (cf. Roberts et al., 2010; Summers et al., 2010). The dichotic configuration used for stimulus presentation limits the energetic masking effects of F2C, because the F1 of the target sentence (presented in the same ear) was lower in frequency and more intense than F2C, and the target F2 was presented in the opposite ear. Therefore, the competitor’s effect must arise primarily through informational masking. Critically, the effect of adding a competitor that matched the harmonic F1+F3 frame was much greater when F2 was tonal than when it was not.

The relatively limited cost of a mismatch between the target F2 and the F1+F3 frame in the absence of F2C suggests that a sine-wave analogue of F2 is capable of conveying phonetic information in a way that can be combined with that carried by the buzz-excited formants. This interpretation presupposes that the intelligibility of the F1+F3 frame alone is relatively low. Consistent with this view, one of our earlier studies found that the mean keyword score for F1+F3 was only 16% for buzz-excited analogues derived from almost continuously voiced sentences spoken by the same talker (Summers et al., 2010, Experiment 1). This outcome is in accord with pilot research using the same sentences as for the current experiment, which indicated that presenting the H1+H3 frame alone would yield scores of ∼20%. In contrast, the dichotic and monaural hybrid conditions (C6 and C8) in the main experiment were associated with keyword scores exceeding 40% and 50%, respectively. These higher levels of intelligibility must reflect the effective integration of phonetic information across harmonic and sine-wave source characteristics.

The results also indicate that T2 tends to lose out when placed in competition with H2C in the context of an H1+H3 frame. Specifically, the cost of competition for T2 is much greater than would be anticipated based on the intelligibility cost of replacing an all-harmonic target with a hybrid target in the absence of the extraneous formant. These findings are consistent with the hypothesis that across-formant grouping is influenced by similarity in source characteristics, such that the target F2 is more easily displaced from the phonetic percept of the sentence when it does not match the acoustic form of the other formants. However, one cannot safely draw this conclusion unless it is possible to demonstrate parallel effects of acoustic source matching and mismatching in the context of a tonal frame.

## Experiment 2

In this experiment, F1 and F3 were always rendered as sine-wave analogues, which have a lower baseline intelligibility than their harmonic counterparts. The target F2 was either generated the same way or by passing a monotonous periodic source through a second-order resonator. Again, when present, the extraneous competitor (F2C) could also take either form. The change to a tonal F1+F3 frame allowed us to assess whether or not the tendency observed in Experiment 1 for a tonal analogue to lose out when competing with a harmonic analogue—irrespective of which one corresponded to the target F2—was genuinely a consequence of grouping by similarity in source characteristics. If so, one would expect the harmonic analogue to lose out to the tonal analogue in the context of a tonal F1+F3 frame.

### Method

Except where described, the same method was used as for Experiment 1. Twenty-four listeners (2 males) passed the training and successfully completed the experiment (mean age = 20.4 years, range = 18.2–34.8); 2 of these listeners were replacements for participants who did not meet the additional criterion of an overall mean score of ≥20% keywords correct in the main session. Previously, all of the listeners had successfully completed at least one speech perception experiment in our laboratory, but none using stimuli derived from the sentences used in the main session. The stimuli for the training session differed from those used in Experiment 1 only in that one half of the sentences were rendered as all-tonal analogues (T1+T2+T3)—that is, sine-wave speech—and for the rest F2 was instead rendered as the output of a buzz-excited resonator (T1+H2+T3).

**Figure 3 fig3:**
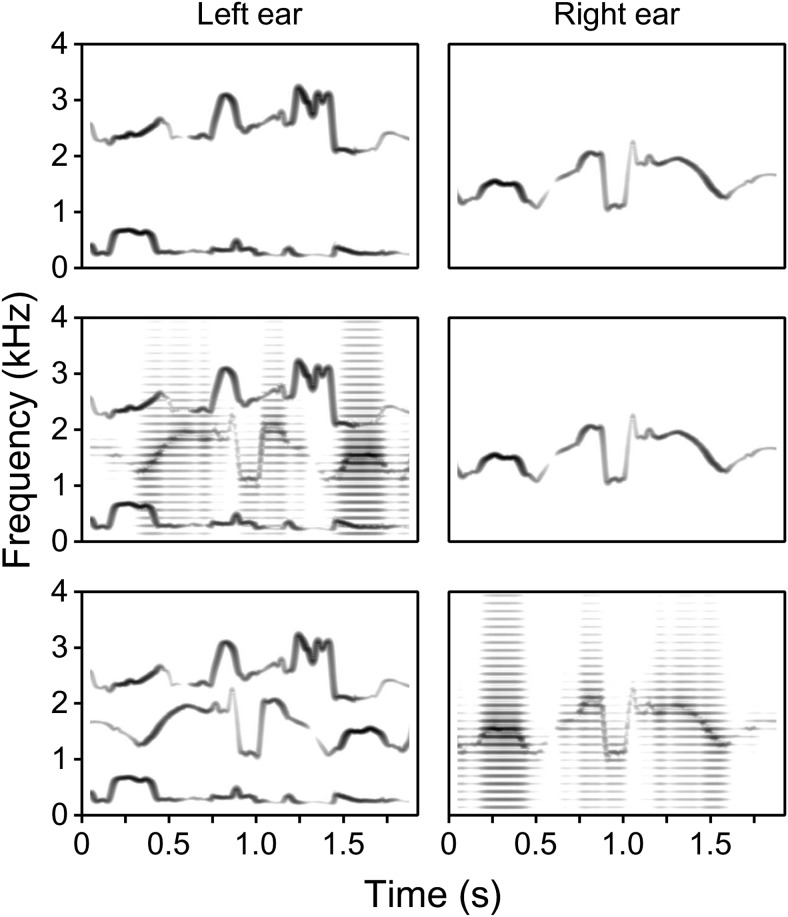
Stimuli for Experiment 2—stimulus spectrograms illustrating three of the dichotic configurations used, derived by narrow-band frequency analysis of synthetic analogues of the example sentence “The man cleaned his shoes.” The left ear receives F1, F3, and (when present) F2C; the right ear receives the target F2. Formants are rendered either as tonal (T) or harmonic (H) analogues. The F1+F3 frame was tonal in all conditions; the F0 frequency of harmonic analogues of F2 and F2C was 140 Hz. The upper, middle, and lower panels illustrate the stimulus configurations (T1+T3; T2), (T1+H2C+T3; T2), and (T1+T2C+T3; H2), respectively. The frequency and amplitude contours of F2C (when present) were time-reversed with respect to the target F2.

The stimuli for the main experiment were derived from a nonoverlapping set of 48 BKB sentences, which were allocated such that there were always 19 or 20 keywords per condition. The harmonic F1+F3 frame shared by all conditions in Experiment 1 was replaced here with a tonal frame (T1+T3), raised to match the RMS power of its harmonic counterpart. Once again, F2 and F2C could be rendered either as the output of a buzz-excited resonator (H2, H2C) or as an RMS-matched time-varying sinusoid (T2, T2C). For consistency with Experiment 1, the sign of the outputs of the resonators corresponding to F2 and F2C was inverted (–). There were 8 conditions in the main session (see Table 2), arranged in an analogous pattern to that used in Experiment 1. Figure 3 illustrates three of the stimulus configurations used—those for C5 (T1+T3; T2) in the top panels, C3 (T1+H2C+T3; T2) in the middle panels, and C4 (T1+T2C+T3; H2) in the bottom panels.[Table-anchor tbl2][Fig-anchor fig3]

### Results

**Figure 4 fig4:**
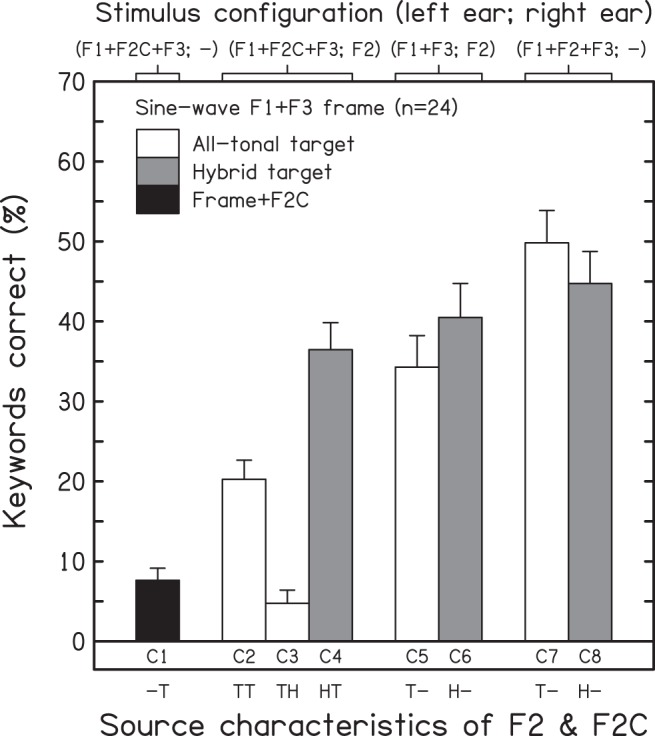
Results for Experiment 2—influence of source characteristics on the effect of competitors (F2Cs) on the intelligibility of synthetic analogues of sentences when the F1+F3 frame was tonal. Mean scores and intersubject standard errors (*n* = 24) are shown for the frame+F2C condition (black bar), the conditions for which all formants of the target speech are tonal analogues (matched, white bars), and the conditions for which the target is a hybrid comprising the tonal F1+F3 frame and a harmonic analogue of F2 (mismatched, gray bars). The top axis indicates which formants were presented to each ear; the bottom axis indicates the source characteristics of F2 and F2C—tonal (T) or harmonic (H). The frequency and amplitude contours of F2C (when present) were time-reversed with respect to the target F2.

Figure 4 shows the mean keyword scores (and intersubject standard errors) across conditions. The black, white, and gray bars indicate the results for the frame+F2C, all-tonal target, and hybrid target conditions, respectively. A one-way ANOVA showed a highly significant effect of condition on intelligibility, *F*(7, 161) = 27.383, *p* < .001, η_p_^2^ = 0.543. Condition C1 (T1+T2C+T3; –) indicated that intelligibility was near floor when F2C was added in the absence of the target F2. Pairwise comparisons indicated that—with the exception of C3—the mean for C1 differed from those for all other conditions, *p* < .001. Again, F2C was not a good surrogate for the target F2 in supporting intelligibility, and performance was best when the target formants were presented monaurally and without competitors (C7 and C8).[Fig-anchor fig4]

A one-way ANOVA restricted to the 5 dichotic cases (C2–C6) also showed a highly significant effect of condition, *F*(4, 92) = 22.205, *p* < .001, η_p_^2^ = 0.491. Adding an F2C to the target formants typically reduced intelligibility, but again the extent of competitor impact depended on the source characteristics of F2 and of F2C. Despite the mismatch in acoustic form, the effect of adding a competitor to an all-tonal target was much greater when F2C was harmonic, C3 versus C5 = 29.5 percentage points, *t*(23) = 7.72, *p* < .001, than when it was tonal, C2 versus C5 = 14.0 percentage points, *t*(23) = 2.93, *p* = .008; this difference was significant, C2 versus C3 = 15.5 percentage points, *t*(23) = 5.63, *p* < .001. Note also that, when F2 was harmonic (i.e., hybrid target), adding a tonal F2C had almost no effect on intelligibility, C4 versus C6 = 4.0 percentage points, *t*(23) = 0.85, *p* = .405, despite the common source characteristics shared by T2C and T1+T3.

The effect of differences in the source characteristics of the target F2 (tonal vs. harmonic) and of its ear of presentation (monaural vs. dichotic) was explored using a two-way ANOVA restricted to the F2C-absent conditions (C5–C8). There was a significant main effect of the ear receiving F2 when the target formants were presented alone, indicating a moderate intelligibility cost of dichotic presentation, mean difference = 9.9 percentage points, *F*(1, 23) = 5.421, *p* = .029, η_p_^2^ = 0.191. However, there was no main effect of the acoustic form of F2, mean difference = 0.6 percentage points, *F*(1, 23) = 0.019, *p* = .893. Despite the suggestion of an interaction between the two factors apparent from a visual inspection of Figure 4—specifically, that the cost of dichotic presentation was greater in the all-tonal case—the interaction term was not significant, *F*(1, 23) = 2.349, *p* = .139.

### Discussion

As might be expected from the higher proportion of target formants presented as sine-wave analogues in Experiment 2, overall intelligibility was lower than for Experiment 1. Nonetheless, in the absence of F2C, the results indicate that listeners can effectively integrate the phonetic information carried by the tonal F1+F3 frame with that carried by the harmonic analogue of F2. Indeed, pilot research indicated that presenting H2 alone to listeners would yield scores below 10%^1^, whereas the scores obtained here in the hybrid target conditions (C6 and C8) exceeded 40%. As for Experiment 1, the intelligibility cost of presenting F1+F3 and F2 to opposite ears in target-only context is broadly in line with the results of our earlier studies. Once again, sentence intelligibility is typically reduced when the target speech is accompanied by an F2C created using the time-reversed frequency and amplitude contours of F2. Critically, the impact of the competitor was greater when it was rendered as a buzz-excited formant, not when it matched the source characteristics of the tonal F1+F3 frame. This outcome is contrary to the argument that similarity in acoustic form plays a major role in across-formant grouping and segregation. Rather, the results of Experiments 1 and 2 taken together suggest that source type and context—not across-formant acoustic similarity—govern the phonetic contribution made by a particular formant.

## General Discussion

The experiments reported here examined the effects of source characteristics, and of differences between formants in their acoustic form, on the across-formant integration of phonetic information. Unlike previous studies, which have focused primarily on across-formant differences in F0 frequency (Bird & Darwin, 1998; Broadbent & Ladefoged, 1957; Cutting, 1976; Darwin, 1981; Gardner et al., 1989; Summers et al., 2010), here differences in the acoustic properties of formants were achieved by rendering them either as the outputs of buzz-excited resonators or as sine-wave analogues. To explore how competition modulates the effects of differences in source characteristics, these effects were compared in the presence and absence of single-formant interferers. There are two striking outcomes of this study. First, in the absence of competition, the integration of phonetic information across formants is not greatly affected by the introduction of radical differences in acoustic source characteristics. This outcome parallels the findings of Darwin (1981) for synthetic vowels and CV syllables. Specifically, the introduction of F0 differences between formants did not affect the perception of phonemic identity under most circumstances, but when the formant ensemble could be interpreted as either of two distinct forms (/ru/ or /li/), F0 differences affected the likelihood of reporting each form. Second, the impact of the competitor formant on intelligibility is not governed by whether its acoustic form matches that shared by F1 and F3. Rather, the phonetic contribution of each alternative version of the second formant (i.e., F2 and F2C) is greater when it is rendered as a buzz-excited formant than as a tonal analogue, irrespective of the source characteristics of the F1+F3 frame. This outcome appears contrary to the idea that informational masking lessens when qualitative differences are introduced between target and masker. The implications of these findings are considered in turn.

It has long been known that sine-wave analogues of formants can carry perceptually useful phonetic information (Bailey et al., 1977; Remez et al., 1981), albeit less effectively than harmonic analogues with the same frequency and amplitude contours. However, to our knowledge, the experiments reported here demonstrate for the first time that intelligible analogues of sentence-length utterances can be created by combining harmonic and tonal renditions of different target formants. This finding adds to the growing body of evidence from studies and simulations of combined acoustic and electroacoustic hearing that phonetic information can be integrated across radically different modes of stimulation (e.g., Qin & Oxenham, 2006; Turner, Gantz, Vidal, Behrens, & Henry, 2004; Verschuur, Boland, Frost, & Constable, 2013). In principle, combined listening is possible for recipients of short-insertion cochlear implants, as this method can preserve remaining low-frequency hearing. Indeed, Turner et al. (2004) showed that three listeners with short-insertion implants and amplified residual hearing performed better than any of their listeners with a traditional implant when listening to target speech accompanied by interfering speech.

Simulations of combined acoustic and electroacoustic listening involve processing the speech in two different ways—usually generating a low-pass filtered version and a vocoded broadband version—and measuring the effect of combining the two signals on intelligibility. For example, Turner et al. (2004) found for normal-hearing listeners that speech reception thresholds in the presence of two-talker babble improved when speech low-pass filtered at 500 Hz was added to 16-channel vocoded speech. Several studies have emphasized the importance of the F0 information (pitch contour) present in the low-pass acoustic signal (Brown & Bacon, 2009; Carroll, Tiaden, & Zeng, 2011; Kong & Carlyon, 2007). However, a recent study has shown that significant benefit can be obtained when a synthetic F1 based on parameters extracted from the original speech, but devoid of pitch-related cues, is combined with simulated electroacoustic hearing (Verschuur et al., 2013). This result is in accord with our findings, because the benefit found can be attributed with greater confidence to an effective integration across frequency regions of the phonetic information presented using distinct modes of stimulation. With improving clinical prospects for short-insertion implantation that retains residual low-frequency hearing, a better understanding of the consequences of mixed-mode listening for speech intelligibility is desirable. The experiments reported here suggest that the integration of phonetic information across modes of stimulation may be greatly affected by the presence of interferers, even in circumstances where masking is primarily informational.

Before considering why a buzz-excited rendition of a formant might dominate over a tonal analogue when they are placed in competition, another aspect of the perception of these hybrid stimuli merits discussion. Sine-wave analogues of speech are usually not heard as speech by listeners on first exposure, but rather as several simultaneous whistles (Bailey et al., 1977; Remez et al., 1981). Once “speech mode” has been engaged by listeners, these sine-wave analogues become intelligible but nonetheless continue to sound like a set of time-varying tones. The occurrence of these two modes of perception—one acoustic and the other phonetic—has been termed “bistability” by Remez, Pardo, Piorkowski, and Rubin (2001). However, given that the two modes are concurrent rather than alternating, this is perhaps better considered as another example of duplex perception, in which one or more acoustic elements contribute simultaneously to two distinct percepts (Liberman, Isenberg, & Rakerd, 1981; Mann & Liberman, 1983; Rand, 1974; see also Bregman, 1990). Neither natural speech nor (for the most part) synthetic speech created by passing a single harmonic source through second-order resonators engages two modes of perception (e.g., Remez et al., 2001). Of course, unlike the components of sine-wave speech, the formants of those stimulus types share a common F0 that encourages their perceptual fusion. Indeed, presenting different formants on different F0s can increase the number of sources heard without necessarily changing the phonetic identity of the stimulus (Broadbent & Ladefoged, 1957; Cutting, 1976).

The hybrid target stimuli used in the study reported here—that is, H1+T2+H3 and T1+H2+T3—are similar to traditional sine-wave speech in that they evoke duplex percepts. However, for the acoustic mode of perception, the multisource character of the stimulus is even more striking owing to the contrast in timbre (complex-tone buzz vs. pure tone) and pitch (constant for buzz, time-varying for pure tone) between the different formant analogues. The results for the competitive conditions examined in the current study also offer some insight into the nature of the phonetic mode of perception. Some researchers have characterized this mode as one in which the usual constraints of auditory scene analysis (Bregman, 1990) are overridden by an abstract form of phonetic coherence when stimulus elements are combined into a speech percept (e.g., Remez, 2003; Remez et al., 1994). Our finding that dissimilarity in acoustic form is not a primary factor in the across-formant integration of phonetic information is indeed consistent with the idea that primitive grouping constraints can be overridden. However, the finding that listeners prefer to integrate the harmonic rather than the sine-wave version of the second formant, irrespective of whether it supports the perception of an intelligible sentence (i.e., F2) or not (i.e., F2C), does not appear to sit comfortably with the notion of phonetic coherence.

As noted above, one of the most striking aspects of our findings is that the buzz analogue dominates over the sinusoidal analogue under competition, even when its integration renders unintelligible a sentence that would otherwise be identified correctly. Why might this be the case? Two obvious ways in which harmonic and sine-wave excitation differ are naturalness and bandwidth. First, harmonic analogues of formants provide a closer approximation than tonal analogues to natural speech. In principle, this difference might be important because of the highly overlearned nature of speech stimuli for adult listeners. Given that differences in naturalness and bandwidth are confounded in the experiments reported here, an account of this kind cannot be ruled out at this point. Note, however, that it would be feasible to explore the role of naturalness of source properties while controlling for any bandwidth effects. This could be done using formant analogues created by passing a variety of excitation sources through second-order resonators, ranging from the relatively natural (buzz or noise sources) to the highly unnatural (components equally spaced in frequency on a log scale, or on a scale related to auditory filter bandwidth—e.g., the Cam scale; Moore, 2012).

Harmonic analogues of formants are wideband stimuli whereas tonal analogues are narrowband. This difference has consequences for their neural encoding in the auditory periphery—and hence for their perceptual properties—that might plausibly influence the informational masking they produce and their effectiveness at signaling phonetic information when placed in competition. These possibilities are considered here. Although the tonal and harmonic counterparts of F2 and F2C were always matched for RMS power in our experiments, wideband signals are typically louder than narrowband signals when matched in this way (e.g., Scharf, 1961; Zwicker, Flottorp, & Stevens, 1957). On that basis, one might speculate that the louder of two versions of a formant will tend to dominate when placed in competition. Note, however, that the loudness difference between a wideband and a narrowband signal is likely to be reduced when most of the energy in the wideband signal is concentrated in a relatively narrow frequency region, as is the case for a buzz-excited formant (cf. Glasberg & Moore, 2010). Moreover, it is well-established that speech perception is fairly insensitive to changes in the relative levels of formants, especially when the more attenuated formants are protected from energetic masking by dichotic presentation (Rand, 1974).

We contend that the tendency for harmonic analogues to dominate over tonal ones when placed in competition is probably a consequence of two bandwidth-related factors. First, there is evidence suggesting that harmonic analogues are more effective at carrying phonetic information than tonal analogues because the former are wideband and the latter are narrowband. Specifically, sine-wave speech becomes more intelligible when high-rate amplitude modulation (AM) is applied to each sinusoid (Carrell & Opie, 1992). This improvement occurs regardless of whether consistent AM rates are applied across formants and so cannot be attributed to grouping based on AM coherence; rather, it must result from the band-widening associated with the addition of modulation side bands (Lewis & Carrell, 2007). Similarly, Souza and Rosen (2009) observed that the intelligibility of three-channel sine-vocoded speech improves as its spectral density increases (i.e., smaller gaps between bands lead to better performance), but this improvement does not depend on the degree of comodulation across bands. Second, we propose on the basis of the results reported here that this underlying difference is magnified by an imbalance in reciprocal informational masking when both versions are present at the same time. Together, these factors result in a substantially greater capacity for the harmonic analogues to transmit phonetic information relative to their tonal counterparts when they are placed in competition.

In the current study, patterns broadly consistent with the band-widening hypothesis can be seen in the results for the conditions without competitors. In Experiment 1 (harmonic frame), there is a main effect of source characteristics in the expected direction across Conditions C5–C8—namely, T2 is less effective than H2 at supporting intelligibility. Although there is no main effect of source characteristics across this set of conditions in Experiment 2 (sine-wave frame), the dichotic case (C5 vs. C6) shows a trend in the expected direction. It is the outcome for the monaural case (C7 vs. C8) that appears discrepant, and this is almost certainly a result of greater energetic masking of F3 by F2 when the lower frequency, more intense formant is wideband than when it is narrowband (cf. Rand, 1974).

Unlike the band-widening experiments described above, the experiments reported here included conditions where alternative versions of a formant were placed in competition—a context for which differences between versions in the transmission efficiency of phonetic information are likely to be critical. Specifically, we propose that dichotic presentation of a wideband (harmonic) and a narrowband (tonal) analogue of a formant leads to asymmetric informational masking, such that the information carried by the harmonic version tends to overwhelm that carried by the tonal version. Hence, in this study, the harmonic version of the second formant becomes dominant in the process of across-frequency integration with the phonetic information carried by F1 and F3, irrespective of whether the harmonic version corresponds to the target F2 or to F2C. It appears that the asymmetry in the informational masking caused by a harmonic formant analogue and its tonal counterpart is also sufficient to override any tendency (if one exists) to group and segregate formants on the basis of similarities and differences in source characteristics.

In conclusion, the experiments reported here indicate that the effects of source characteristics on the phonetic contributions made by individual formants in an ensemble are governed by type and context, rather than by target-masker similarity. These findings help to refine our understanding of how phonetic information is carried by formants and combined across them, particularly in circumstances where extraneous formants are present and act primarily as informational maskers. Aside from their theoretical interest, these findings may also have implications for enhancing effective mixed-mode listening in clinical contexts.

## Supplementary Material

10.1037/xhp0000038.supp

## Figures and Tables

**Table 1 tbl1:** Stimulus Properties for the Conditions Used in Experiment 1 (Main Session)

Condition	Stimulus configuration (left ear)	Stimulus configuration (right ear)
C1	H1+H2C+H3	—
C2	H1+H2C+H3	H2
C3	H1+T2C+H3	H2
C4	H1+H2C+H3	T2
C5	H1+H3	H2
C6	H1+H3	T2
C7	H1+H2+H3	—
C8	H1+T2+H3	—
*Note.* H and T denote harmonic and tonal formant analogues, respectively. The F1+F3 frame was harmonic in all conditions. The F0 frequency of the Rosenberg source for the harmonic analogues of F1, F2, F3, and F2C was always 140 Hz.		

**Table 2 tbl2:** Stimulus Properties for the Conditions Used in Experiment 2 (Main Session)

Condition	Stimulus configuration (left ear)	Stimulus configuration (right ear)
C1	T1+T2C+T3	—
C2	T1+T2C+T3	T2
C3	T1+H2C+T3	T2
C4	T1+T2C+T3	H2
C5	T1+T3	T2
C6	T1+T3	H2
C7	T1+T2+T3	—
C8	T1+H2+T3	—
*Note.* T and H denote tonal and harmonic formant analogues, respectively. The F1+F3 frame was tonal in all conditions. The F0 frequency of the Rosenberg source for the harmonic analogues of F2 and F2C was always 140 Hz.		

## Footnotes

1 Note that the intelligibility of an isolated F2 analogue is far lower than would be the case for a sentence-length natural utterance band-pass filtered around 1,500 Hz (for a male talker). This is because it is not possible to isolate F2 in a natural utterance; the band-pass version would inevitably carry useful information about the movements of F1, and of F3 and the higher formants. Indeed, natural speech can sometimes remain intelligible even when passed through extremely narrow spectral slits (Warren, Riener, Bashford, & Brubaker, 1995). This point is also relevant to the intelligibility of the F1+F3 frame used in Experiment 1.
