# Supplementary material for: Acoustic Source Characteristics, Across-Formant Integration, and Speech Intelligibility Under Competitive Conditions
Source: J Exp Psychol Hum Percept Perform. 2015 Mar 9;41(3):680–91. doi: 10.1037/xhp0000038 (PMC4445382; doi:10.1037/xhp0000038)
Supplement: Supplementary file 1 [file xhp-XHP-2014-0232-Roberts_Summers_Bailey_2015_Additional_Content.pdf]

Additional content for *"Acoustic source characteristics, across-formant integration, and speech intelligibility under competitive conditions,"* by Brian Roberts, Robert J. Summers, and Peter J. Bailey.

The audio files accompanying this document illustrate the target-only conditions (i.e., no competitors present) for the two experiments described in the main article. These examples differ from their counterparts in conditions C7 and C8 of these experiments only in that they are configured for diotic (instead of monaural) presentation.

All the examples are synthetic three-formant analogues of speech (F1+F2+F3); each formant can be either buzz-excited (harmonic source, H) or sine-wave (tonal source, T). The harmonic source has a constant F0 of 140 Hz.

Each file name specifies first the source characteristics of F1+F3 (Harmonic or Tonal) and second whether the source characteristics of F2 are the same as (Standard) or different from (Hybrid) those of F1+F3. For the hybrid cases, the source characteristics of F2 are shown in bold in the table below.

The unusual speech quality for these stimuli is a consequence not only of the particular source characteristics used, but also the simplicity of the synthesis strategy. When comparing the examples, note how clearly one can hear the two distinct acoustic sources when listening to the hybrid stimuli. Nonetheless, these hybrid materials remain intelligible.

**Filename**	**Acoustic Sources**	**Sentence**
Harmonic_Standard_bkbq0408p1.wav<br>Harmonic_Hybrid_bkbq0408p1.wav	H1+H2+H3<br>H1+**T2**+H3	"Lemons grow on trees"
Harmonic_ Standard_bkbq0604p1.wav<br>Harmonic_Hybrid_bkbq0604p1.wav	H1+H2+H3<br>H1+**T2**+H3	"Men wear long trousers"
Tonal_ Standard_bkbq0310p1.wav<br>Tonal_Hybrid_bkbq0310p1.wav	T1+T2+T3<br>T1+**H2**+T3	"The man cleaned his shoes"
Tonal_ Standard_bkbq1008p1.wav<br>Tonal_Hybrid_bkbq1008p1.wav	T1+T2+T3<br>T1+**H2**+T3	"Someone's crossing the road"

This document was created on 28th January 2015
